# Solution Structure and NMR Chemical Shift Perturbations of the Arabidopsis BCCP1 Identify Intersubunit Interactions Potentially Involved in the Assembly of the Heteromeric Acetyl‐CoA Carboxylase

**DOI:** 10.1002/pld3.70057

**Published:** 2025-03-21

**Authors:** Kiran‐Kumar Shivaiah, Ganesh P. Subedi, Adam W. Barb, Basil J. Nikolau

**Affiliations:** ^1^ Roy J. Carver Department of Biochemistry, Biophysics, and Molecular Biology Iowa State University Ames Iowa USA; ^2^ Center for Biorenewable Chemicals Iowa State University Ames Iowa USA; ^3^ Center for Metabolic Biology Iowa State University Ames Iowa USA; ^4^ Department of Biochemistry and Molecular Biology, DOE‐Plant Research Laboratory Michigan State University East Lansing Michigan USA; ^5^ Department of Biochemistry and Molecular Biology University of Georgia Athens Georgia USA

**Keywords:** biotin attachment domain–containing proteins, biotin carboxyl carrier protein, biotin carboxylase, chemical shift perturbation, heteromeric acetyl‐CoA carboxylase, NMR

## Abstract

Biotin carboxyl carrier protein (BCCP) is a subunit of the heteromeric acetyl‐CoA carboxylase (htACCase), and it chemically links the two half‐reactions that constitute the formation of malonyl‐CoA from acetyl‐CoA, a critical reaction in fatty acid biosynthesis. Because plants are a major source of edible fats and oils, it is important to understand the structural organization of the plant htACCase, relative to its potential to regulate fatty acid biosynthesis in plant plastids. Moreover, unique to the plant htACCase, noncatalytic subunits called biotin attachment domain–containing (BADC) proteins are important in the assembly of the holoenzyme, and they specifically interact with the bcCP and the biotin carboxylase (BC) subunits. We report herein NMR structural studies of the Arabidopsis BCCP isozymes (bcCP1 and BCCP2). We calculated the structure of C‐terminal domain of BCCP1 (K_200_‐P_280_) and explored structural changes in the BCCP1 protein upon its interactions with bc and BADC. The chemical shift perturbation experiments identified potential surface residues on the BCCP1 protein that may facilitate physical interactions between BC and BADC proteins. These studies indicate that the BADC protein interacts with a “thumb”‐like protrusion, which is a common structural feature of the bacterial and plant bcCPs, and thereby acts as a potential “cap” to facilitate the assembly of a BC–BCCP–BADC complex.

## 1 Introduction

Biotin‐containing enzymes occur universally in all organisms, and they catalyze carboxylation, decarboxylation, or transcarboxylation reactions (Cronan and Waldrop 2002; Nikolau et al. 2003; Tong 2013). Typical of biotinylated enzymes, acetyl‐CoA carboxylase (ACCase) (EC 6.4.1.2) catalyzes a reaction that is the sum of two half reactions. The first half reaction, catalyzed by the biotin carboxylase (bc) functionality, is the ATP‐dependent carboxylation of the biotin prosthetic group, which is carried by the biotin carboxy‐carrier protein (BCCP). The second half reaction is catalyzed by the carboxyltransferase (CT) functionality, which is the transfer of the carboxyl group from carboxy‐biotin intermediate to acetyl‐CoA and thereby forming malonyl‐CoA (Nikolau et al. 2003; Waldrop et al. 2012; Tong 2013; Tong 2017; Cronan 2021). The formation of malonyl‐CoA is critical in the biosynthesis of fatty acids (McCarthy and Hardie 1984) and polyketides (Hopwood and Sherman 1990).

Two distinct forms of ACCase are present in most flowering plants, and each is located in different subcellular compartments (Sasaki et al. 1993; Nikolau et al. 2003). The cytosolic ACCase is a homomeric dimer and all functional components (i.e., bc, BCCP, and CT) occur as distinct recognizable domains within a single polypeptide chain (López‐Casillas et al. 1989; Gornicki et al. 1993; Konishi et al. 1996; Nikolau et al. 2003; Al‐Feel et al. 2006). This structural organization is analogous to the ACCase that also occurs in animals and fungi (Brownsey et al. 2006; Wakil and Abu‐Elheiga 2008; Tong 2013). In most plants, with the exception of the Poaceae, the plastids contain a heteromeric ACCase (htACCase) and the three catalytic functionalities (bc, BCCP, and CT) are expressed as separate subunits (Nikolau et al. 2003; Sasaki and Nagano 2004). The bacterial ACCase from *Escherichia coli* can be considered the archetypal of the htACCase enzyme, consisting of separate subunits (Dimroth et al. 1970; Fall and Vagelos 1972; Guchhait et al. 1974). The structure of these subunits has been determined, demonstrating the subcomplexes that are assembled to form the holoenzyme (Mochalkin et al. 2008; Broussard et al. 2013b; Tong 2013; Cronan 2021).

**FIGURE 1:**
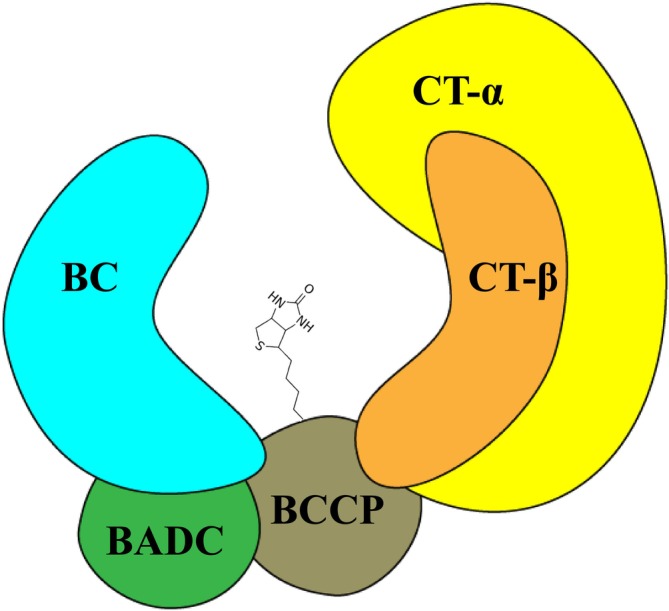
A schematic representation of the quaternary organization of catalytic subunits (bc, bcCP, CT‐α and CT‐β) and noncatalytic subunit (BADC) of the heteromeric acetyl‐CoA carboxylase. The biotin prosthetic group that is covalently bound to the bcCP subunit oscillates between the bc and CT catalytic sites to catalyze the carboxylation of acetyl‐CoA, forming malonyl‐CoA.

Although the plant htACCase is less well characterized, it has become apparent that this enzyme has a different quaternary organization from the bacterial ACCase. As shown in Figure 1, this conclusion is based on the finding that in addition to the homologous catalytic subunits (i.e., bc, BCCP, CT‐α, and CT‐β) that form the core of the plant enzyme, the isolated megadalton htACCase complex also contains noncatalytic subunits, specifically, the biotin attachment domain–containing (BADC) protein (Olinares et al. 2010). An additional noncatalytic component of this enzyme, which is not shown in Figure 1, is the global metabolic regulator protein, PII (Feria Bourrellier et al. 2010).

In Arabidopsis, as is typical of most plants, the bc subunit (At5g35360), one of the CT subunits (CT‐α; At2g38040), and the two BCCP subunit isoforms (BCCP1; At5g16390 and BCCP2; At5g15530) are encoded by nuclear located genes, whereas, the CT‐β subunit (Atcg00500) is encoded by the plastome (Choi et al. 1995; Ke et al. 1997; Thelen et al. 2001). Genetic studies have revealed a unidirectional redundancy between BCCP1 and BCCP2; namely, whereas the expression of the former is absolutely essential for viability, the expression of the latter is not required (Li et al. 2011). The nonviability of plants lacking a functional *BCCP1* gene occurs during very early stages of seed development (Li et al. 2011), and this is despite the high levels of *BCCP2* expression that occurs during seed development (Thelen et al. 2001). Hence, BCCP2 appears to be redundant for growth, development, and propagation.

Additionally, the Arabidopsis genome encodes three BADC isoforms: BADC1 (At3g56130), BADC2 (At1g52670), and BADC3 (At3g15690), and although they share 25%–30% sequence identity with the BCCP subunits, they are not biotinylated, as they lack the “AMKL” biotinylation motif sequence (Salie and Thelen 2016; Keereetaweep et al. 2018; Shivaiah et al. 2020). In vivo studies have suggested that BADC proteins regulate ACCase activity by acting as inhibitors (Salie et al. 2016; Keereetaweep et al. 2018). However, more detailed in vitro reconstitution experiments, coupled with genetic studies, have revealed that the BADC proteins act as a “glue,” facilitating the assembly of a bc‐BCCP subcomplex, which also facilitates the assembly of the active htACCase complex that contains the CT‐α/CT‐β subcomplex (Shivaiah et al. 2020). This is consistent with findings that certain acyl‐CoA carboxylases from bacteria utilize a noncatalytic subunit as a “glue” to assemble the holoenzyme complex and activate catalysis (Gago et al. 2006; Gago et al. 2011; Shivaiah et al. 2021). More specifically, the BADC proteins interact with the N‐terminal region of the BCCP subunits, which is a Ala‐, Pro‐ and Ser‐rich sequence, which is typical of proteins that have a disordered structure (Campen et al. 2008; Uversky and Dunker 2010). In the current study, we have employed NMR structure calculations and chemical shift perturbation studies to calculate the structure of C‐terminal domain of bcCP1 and explored structural changes in the bcCP1 protein upon its potential interactions with BC and BADC. The chemical shift perturbation experiments identified surface residues on the BCCP1 protein that may facilitate the physical interactions between BC and BADC proteins.

## 2 Materials and Methods

### 2.1 Heterologous Protein Expression Vectors

DNA sequences coding for the BCCP1 and BCCP2 ORFs were subcloned from already described vectors (Li et al. 2011). The transit chloroplast‐targeting peptides of these plant proteins were identified by using the TargetP tool (www.cbs.dt/services/TagetP) (Nielsen et al. 1997; Emanuelsson et al. 2000). Additionally, BLASTP (Altschul et al. 1990; Madeira et al. 2022) and the multiple sequence alignment tool from Invitrogen Technologies (Grand Island, New York) were used to compare the plant BCCP1 and BCCP2 proteins with bacterial homologs that do not carry such transit peptides. Based on these analyses, the sequences that encode the initial 80 and 87 residues of the full‐length BCCP1 and BCCP2 protein (the putative transit chloroplast‐targeting peptides) were genetically excised. The ORF sequences encoding the mature BCCP1_81–280_ and mature BCCP2_88–255_ proteins were cloned into a modified pET30f expression vector (Jing et al. 2018).

The ORF sequence that encodes the C‐terminal 81‐residues of the BCCP1 protein (i.e., BCCP1_200–280_) and C‐terminal 81‐residues of the BCCP2 protein (i.e., BCCP2_175–255_) were cloned into the pET30f expression vector. The resulting expressed proteins were called the Arabidopsis C‐terminal domain of BCCP1 and C‐terminal domain of BCCP2, respectively. The amino acids in the NMR assignments are numbered starting from the mature protein (P1–P200), excluding the 80 residues from the transit peptide (Figure S1).

### 2.2 Expression and Purification of BCCP1 and BCCP2 Proteins

The recombinant pET30f‐derivative vectors for expressing the mature BCCP1 (BCCP1_81–280_), mature BCCP2 (BCCP2_88–255_), C‐terminal domain of BCCP1, and C‐terminal domain of BCCP2 proteins were carried by *E. coli* strain, BL21 (DE3). Expression cultures were initiated by inoculating with a single colony, a 5 mL of M9 minimal media containing 1 g/L ^15^N‐ammonium chloride, 2 g/L ^13^C‐D‐glucose, and 50 μg/mL of kanamycin. Following overnight incubation at 37°C with agitation, the entire culture was used as the inoculum to initiate a 500‐mL culture of the identical medium composition. To produce ^15^N‐labeled and ^13^C‐labeled proteins, these media were supplemented with ^15^N‐ammonium chloride and D‐glucose‐[U‐^13^C6] (2 g/L) (Cambridge Isotope Laboratories Inc., Massachusetts). The culture was incubated at 37°C, until it reached an OD at 600 nm between 0.6 and 0.8, and protein expression was induced by adjusting the culture to 0.4 mM IPTG. The culture was incubated for an additional 18–24 h at 22°C. Cells were harvested by centrifugation, and the resulting pellet was flash frozen with liquid nitrogen, before storage at −80°C.

The cell pellet was resuspended in a buffer consisting of 20 mM Tris‐Cl, pH 8.0, 500 mM NaCl, and 5 mM imidazole and Pierce EDTA‐free protease inhibitor cocktail (one tablet per 50 mL of buffer) (Thermo Scientific, Waltham, Massachusetts). The cell suspension was sonicated on ice with 7–10 bursts, each of 15‐s duration, and each was followed by a 1 min of cooling. The sonicated lysate was centrifuged at 20,000 *g* for 30 min to remove membranes and cell debris. The supernatant was recovered and filtered with a 0.45‐μm nitrocellulose filter (Corning Lifesciences, Corning, New York, United States). The filtrate was loaded onto a Ni‐NTA agarose column (5 mL bed volume) that had been pre‐equilibrated with 20 mM Tris‐Cl, pH 8.0, 500 mM NaCl, and 5 mM imidazole. Nonbound proteins were removed by washing the column with the same column buffer, and His‐tagged proteins that were bound to the column were eluted with a buffer consisting of 20 mM Tris‐Cl, pH 8.0, 500 mM NaCl, and 120 mM imidazole. The eluent was dialyzed against 20 mM phosphate buffer, pH 7.2, containing 150 mM NaCl, 10% (v/v) glycerol and 2 mM DTT. The purified protein preparations were concentrated using Amicon Ultra‐15 centrifugation columns (MilliporeSigma, Billerica, Massachusetts).

In order to validate whether N‐terminal 6X His‐tag influences the chemical environment of BCCP1, the Arabidopsis C‐terminal domain of BCCP1 was expressed and purified as described, and after purification, the N‐terminal 6X His‐tag was proteolytically removed using an established protocol (Tropea et al. 2009). Specifically, the purified BCCP1 C‐terminal protein was incubated overnight at 16°C with TEV protease (1 μg of TEV protease per 50 μg of the purified BCCP1 C‐terminal protein). The digested sample was passed through the Ni‐NTA column pre‐equilibrated with 50 mM phosphate buffer, pH 7.4, containing 150 mM NaCl and 10% glycerol, which retained the hydrolyzed 6X His‐tag and the undigested 6X His‐tagged C‐terminal domain of BCCP1. The flow‐through fractions containing the C‐terminal domain BCCP1 protein without the 6X His‐tagged C‐terminal domain was recovered and dialyzed against 20 mM phosphate buffer, pH 7.2, containing 150 mM NaCl, 10% (v/v) glycerol, and 2 mM DTT, and concentrated using an Amicon Ultra‐15 centrifugation column (MilliporeSigma, Billerica, Massachusetts). C‐terminal domain of bcCP1 with and without His‐tag was subjected to ^1^H‐^15^N HSQC experiments. The overlay of the spectra revealed that presence of the 6X His‐tag had no detectable effect on the resulting HSQC spectra (data not shown).

### 2.3 Cloning and Expression of bc and BADC3 Proteins

BADC3_(A55‐Q263)_ and bc_(C71‐V537)_ proteins were subcloned into pET30f from previously published expression vectors (Sun et al. 1997; Shivaiah et al. 2020). These proteins were expressed and purified using procedures described for purifying the BCCP1 protein. Copurification of mature BCCP1 with BADC3 in the presence and absence of BC was conducted as previously described (Shivaiah et al. 2020).

### 2.4 NMR Spectroscopy

All NMR spectra were collected at 303 K, using either Bruker Avance II 700 or Bruker Avance III 800 spectrometers equipped with 5‐mm TCI cryoprobes and housed at Iowa State University's Biomolecular Nuclear Magnetic Resonance Facility. The NMR spectra were collected with 600‐μL samples of homogenously ^15^N‐ and ^13^C‐labeled proteins or with homogeneously ^15^N‐labeled proteins. The protein solutions were in 20 mM phosphate buffer, pH 7.2, containing 150 mM NaCl, 10% (v/v) glycerol, and 2 mM DTT prepared in 10% (v/v) ^2^H_2_O. The two‐dimensional (2D) ^1^H‐^15^N HSQC and ^1^H‐^13^C HSQC and three‐dimensional (3D) CBCA (CO)NH, CBCANH, HNCA, HN (CO)CA, HNHA, HNCO, and HCCH‐TOCSY data were acquired using a Bruker 700 spectrometer, whereas 3D‐C (CO)NH, H (CCO)NH, and HBHA (CO)NH data were acquired using the Bruker 800 spectrometer. All the spectral data were processed using TopSpin software obtained from Bruker.

The analyses of the spectral data and the chemical shift assignment were conducted using SPARKY (Lee eat al., 2015). The sequential backbone assignment of the protein was calculated using [^15^N,^1^H] HSQC, CBCA (CO)NH, CBCANH, HNCA, and spectra HN (CO)CA. The automated assignment feature in SPARKY, called PINE, was used in corroborating the manual assignments (Lee et al. 2009). The sidechain assignments were achieved using 3D‐C (CO)NH, H (CCO)NH, and HBHA (CO)NH and further by using [^13^C, ^1^H] HSQC and HCCH‐TOCSY.

CS‐Rosetta was used to determine the chemical shift values to select the fragments from Protein Data Bank (PDB) in combination with the Rosetta Monte Carlo assembly and relaxation methods (Shen et al. 2008; Shen et al. 2009; Shen et al. 2010; Lange et al. 2012).

The chemical shift perturbation values (ΔδAvg HN) for the backbone amide bonds were evaluated by using NMRSPARKY program. The equation used for this calculation was as follows:

$$\text{ΔδAvg}\;\mathrm{HN}=\sqrt{{\left(\mathrm{\Delta H}\right)}^{2}+{\left(\frac{\mathrm{\Delta N}}{2}\right)}^{2}}$$

where, ΔH and ΔN are the chemical shift changes of the amide ^1^H and ^15^N in parts per million, induced by conformational changes upon protein–protein interactions.

## 3 Results and Discussion

**FIGURE 2:**
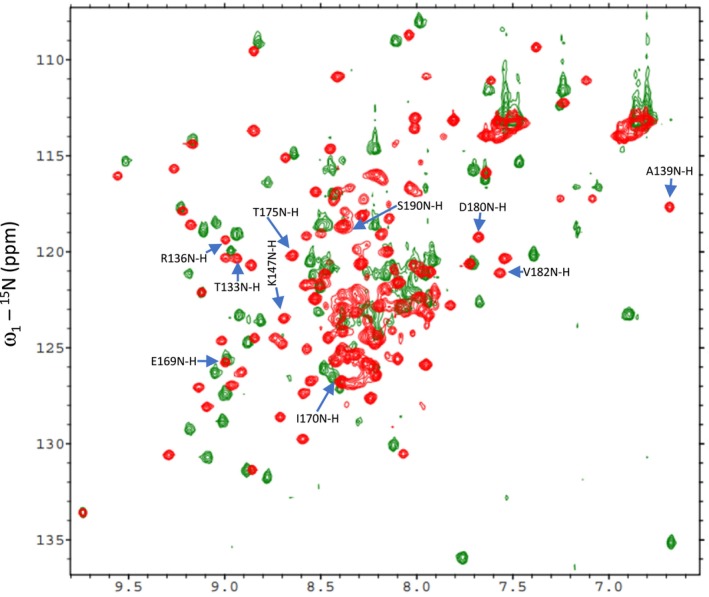
Overlay of ^1^H‐^15^N HSQC spectra of the mature bcCP1 (red) and mature bcCP2 (green) proteins. More than 50% of the amide peaks are overlapping at the center of the spectra indicating the presence of unstructured or flexible regions in the two proteins.

**FIGURE 3:**
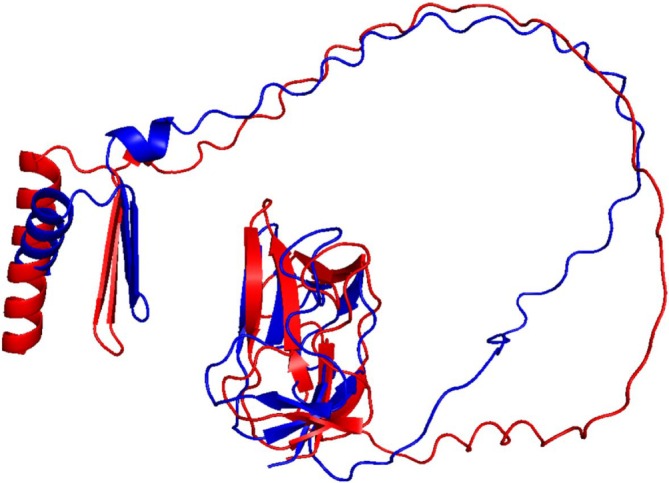
Comparison of Alphafold2 predicted structures of mature bcCP1 (red) and mature bcCP2 (blue) visualized with PyMOL (RRID:SCR_000305).

The two BCCP genes of Arabidopsis express proteins of 280 (BCCP1) and 255 (BCCP2) residues, which share a high degree of sequence homology (Figure S1). Each of these proteins is initially expressed with an 81‐ and 87‐residue chloroplast‐targeting signal peptide, which is removed during chloroplast import, resulting in the accumulation of 199‐ and 168‐residue mature proteins (Thelen et al. 2001; Li et al. 2011). The C‐terminal 81‐residues of these two proteins contain the site of biotinylation, and they share a high level of sequence homology (80% identity) (Figure S1). Based on the amino acid sequences of the two Arabidopsis BCCP isoforms, 188 and 150 ^1^H‐^15^N HSQC peaks were expected from the mature BCCP1 and mature BCCP2, respectively; these include the Nδ‐Hδ2/Nε‐Hε2 peaks from the amide groups of Asn/Gln side chains. However, only about 50% of the expected number of discreet ^1^H‐^15^N HSQC peaks were detectable in the HSQC spectra of these proteins (Figure 2). Furthermore, these ^1^H‐^15^N HSQC spectra have a large number of unresolvable amide peaks in the middle of the spectra, which indicates the presence of unstructured or flexible regions in both of these proteins. This structural flexibility is expected for proteins that contain Ala‐, Ser‐, and Pro‐rich regions (Miles et al. 1987; Yao et al. 1999; Uversky and Dunker 2010), which is the case for both BCCP1 and BCCP2 sequences, particularly in the N‐terminal half of the mature proteins. Indeed, the lack of NOE peaks in the N‐terminal region of the BCCP of the transcarboxylase from *Propionibacterium shermanii* has previously been attributed to the high degree of flexibility associated with this region of this enzyme subunit (Reddy et al. 2000). Such Ala‐, Ser‐, and Pro‐rich regions are often found with intrinsically disordered proteins, which do not form stable secondary structural motifs (Perham 2000; Oldfield and Dunker 2014; Uversky 2016; Malagrinò et al. 2022). Furthermore, Alphafold2 (Jumper et al. 2021) generated predicted models of the mature BCCP1 and mature BCCP2, indicating that the C‐terminal portion of these proteins form well‐structured domains that appear similar to each other (Figure 3), reflecting the high degree of sequence similarity that is shared by these two domains. However, the N‐terminal portion of both these proteins shares less homology and is predicted to be primarily unstructured, with the exception of an α‐helix and a β‐sheet in the N‐terminal segment of both of these proteins (Figure 3).

**FIGURE 4:**
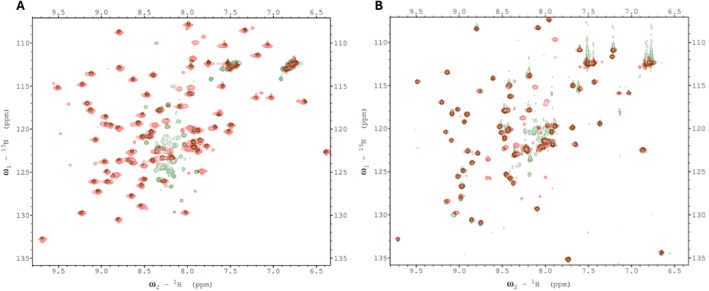
(A) Overlay of ^1^H‐^15^N HSQC spectra of the mature bcCP1 (green) and the C‐terminal domain of bcCP1 (red). (B) Overlay of ^1^H‐^15^N HSQC spectra of the mature BCCP2 (green) and the C‐terminal domain of BCCP2 (red). All resolvable amide peaks that are detectable in the spectra obtained with the mature BCCP1 or BCCP2 proteins (green) are also detectable in the spectra obtained with the C‐terminal domains of the BCCP1 or BCCP2 proteins (red), indicating that the N‐terminal domain of the BCCP proteins is unstructured.

We verified these predictions, by conducting ^1^H‐^15^N HSQC experiments with ^15^N‐labeled, C‐terminal domains of BCCP1 and BCCP2 that lacked the N‐terminal 199 and 174 residues of these proteins, respectively. Comparing the spectra of the mature and the C‐terminal domains of BCCP1 or BCCP2 confirmed that the well‐resolved HSQC peaks observed with the mature proteins are generated by residues located at the C‐terminal domain of either BCCP1 or BCCP2, respectively (Figure 4). These results substantiate the hypothesis that the N‐terminal domain contributes to the nonresolved middle region of the HSQC spectra generated by the mature BCCP1 and BCCP2 proteins (Figure 2), validating that the chemical environment of the amino acids from this region of these proteins is devoid of a stable secondary and tertiary structure. Moreover, no experimental evidence was found in the NMR spectra for the occurrence of the secondary structural features in the N‐terminal region of the mature BCCP1 or BCCP2 that are predicted by Alphafold2 (Figure 3); this is despite the fact that the prediction accuracy was greater than 90% for the α‐helix secondary structure.

### 3.1 Calculation of the Structure of C‐Terminal Domain of the Arabidopsis BCCP1

**FIGURE 5:**
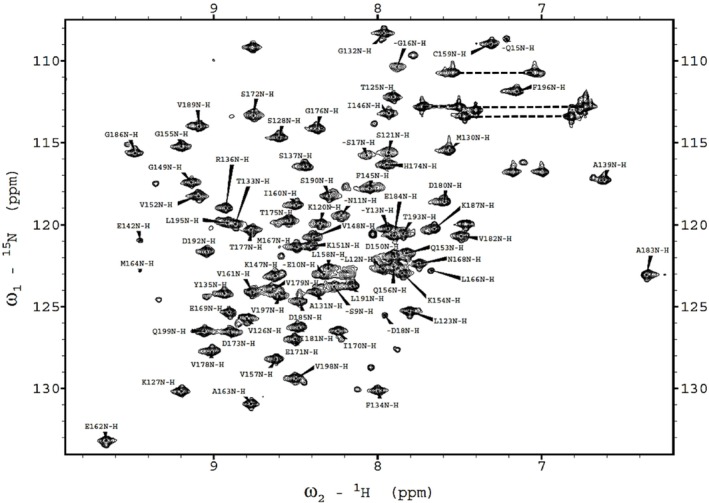
Two‐dimensional ^1^H‐^15^N HSQC spectra of C‐terminal domain of BCCP1 (pH = 7.2, T = 25.0°C, 90% ^1^H_2_O/10% ^2^H_2_O). The spectrum shows the N‐H correlations of all the residues, except the 10 Pro residues, and Gly141. NMR data was processed using Bruker TopSpin software and the analyzed using NMRSPARKY program (Lee et al. 2015).

We collected triple‐resonance NMR spectra to assign the backbone resonances, including CBCA (CO)NH, CBCANH, HNCA, HN (CO)CA, HBHA (CO)NH, HCCH‐TOCSY, C (CO)NH, H (CCO)NH, and HBHA (CO)NH. We evaluated NMR data with the SPARKY program (32) hosted by NMRFAM to establish backbone and sidechain assignments in the gathered NMR data. This resulted in the assignment of chemical shift values for 81 residues, out of the 100 residues of the expressed C‐terminal domain of BCCP1 (Figure 5). Additionally, 96% of Cα, 95% of Cβ, and 84% of CO resonances were assigned. These assigned chemical shift data have been deposited in the BioMagResBank database (http://www.bmrb.wisc.edu), as BMRB ID 52087, and with wwPDB (https://www.wwpdb.org) as accession number PDBDEV_00000372.

**FIGURE 6:**
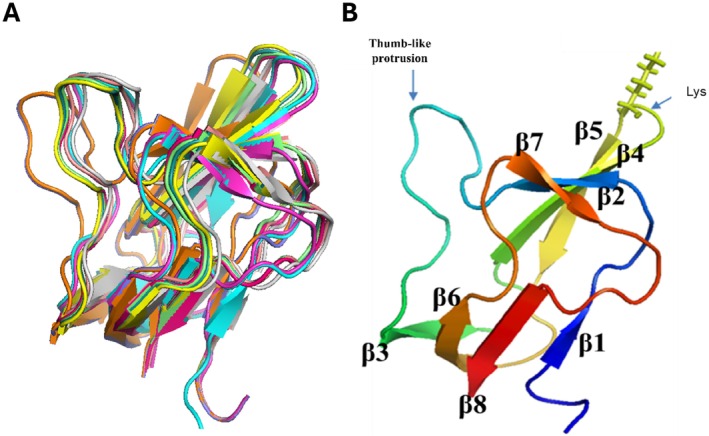
(A) Superimposition of the 10 best structures of C‐terminal domain of BCCP1 predicted by CS‐Rosetta. The Cα‐RMSD against lowest energy structure was 0.33 ± 0.16 Å. (B) Ribbon diagram representation of CS‐Rosetta generated structure of the C‐terminal domain of BCCP1 showing the positions of the biotinylated Lys residue and the thumb‐like hairpin loop region.

Using the assigned chemical shift values, the structure of the C‐terminal domain of the Arabidopsis BCCP1 was determined using chemical shift‐Rosetta (CS‐Rosetta) (Shen et al. 2008; Shen et al. 2009; Shen et al. 2010; Lange et al. 2012). The first step in this calculation utilized chemical shift values of the ^13^Cα, ^13^Cβ, ^13^C′, ^15^N, ^1^HA, and ^1^HN and the known amino acid sequence of this protein to select homologous polypeptide fragments from PDB (Berman et al. 2000). Subsequently, these smaller sets of fragments were utilized with the Rosetta‐based Monte Carlo assembly method (Metropolis and Ulam 1949) to generate 3000 predicted structures of the C‐terminal domain of BCCP1. The final convergence was decided based on how well the coordinates of the lowest energy structures agree with one another. By selecting Cα RMSD of < 2 Å, the structure calculations converged to the 10 lowest energy models (Figure 6A), with an average Cα RMSD of 0.33 ± 0.16 Å (Table S1 and Figure S2). The quality of the calculated models was further tested using the protein structure validation software suite, PSVS (https://montelionelab.chem.rpi.edu/PSVS/PSVS/). PSVS integrates multiple structure validation tools, these being MolProbity (Williams et al. 2018), Verify3D (Bowie et al. 1991; Lüthy et al. 1992), Prossa II (Sippl 1993; Wiederstein and Sippl 2007), RPF 2 (Huang et al. 2005), PROCHECK (Laskowski et al. 1996), and the PDB validation software and several other structure‐validation tools (https://sw‐tools.rcsb.org/apps/VAL/). MolProbity demonstrated that 100% of Psi (ψ)–Phi (φ) torsion angle pairs are in the most favored region of the Ramachandran plot. Additionally, PROCHECK indicates that > 90% of Psi (ψ)–Phi (φ) torsion angle pairs are in the most favored region, and the other ~10% of torsion angle pairs are in the allowed region of the Ramachandran plot.

Figure 6B shows the resulting convergence structure of the C‐terminal domain of the Arabidopsis BCCP1, which consist of eight β strands, located between residues T125‐K127 (β1), G132‐Y135 (β2), G149‐Q153 (β3), L158‐A163 (β4), L166‐E171 (β5), T175‐D180 (β6), G186‐V189 (β7), and L195‐Q199 (β8), which form two β‐sheet domains. One β‐sheet domain is assembled by H‐bond interactions among β1, β3, β6, and β8 strands, and the second β‐sheet domain is assembled by H‐bond interactions among β2, β4, β5, and β7 strands. The biotinylated Lys165 residue is located in the hairpin turn between β4 and β5.

### 3.2 Comparisons to Homologous BCCP Domain Structures

**FIGURE 7:**
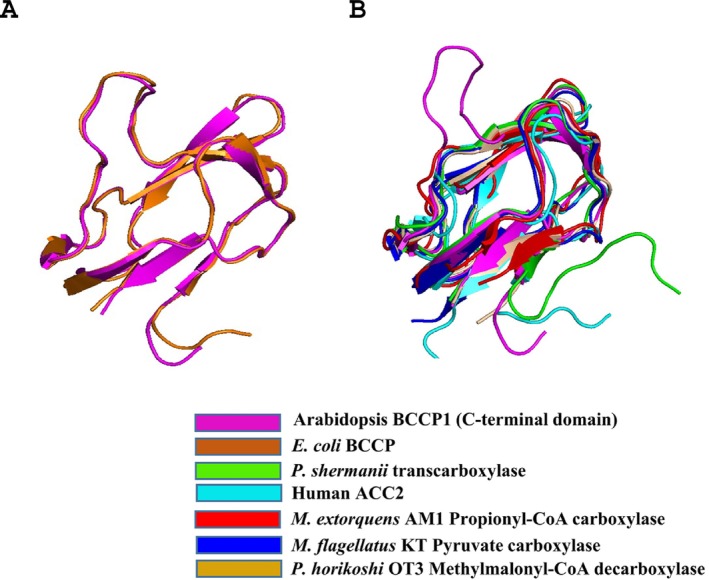
(A) Superposition of the structure of the C‐terminal domain of the *E. coli* BCCP (orange, PDB: 4HR7, Broussard et al. 2015) with the C‐terminal domain of the Arabidopsis BCCP1 (magenta), from the current study. (B) Superposition of the structures of the BCCP domains of the human ACC2 (cyan, PDB: 2KCC, Lee et al. 2008), 1.3S subunit of transcarboxylase from *Propionibacterium shermanii* (green, PDB: 1DCZ, Reddy et al. 1998), propionyl‐CoA carboxylase from *Methylorubrum extorquens* AM1 (red, PDB: 6YBP, Scheffen et al. 2021), pyruvate carboxylase from *Methylobacillus flagellatus* KT (blue, PDB: 5KS8, (Choi et al. 2016), methylmalonyl‐CoA decarboxylase from *Pyrococcus horikoshii* OT3 (wheat, PDB ID: 2D5D, Bagautdinov et al. 2008) and the structure of the C‐terminal domain of the Arabidopsis BCCP1 (Magenta), determined in this study.

The overall protein fold of the C‐terminal domain of Arabidopsis BCCP1 shows a two‐fold quasisymmetry, comprising a flattened β‐barrel formed by two four‐stranded β‐sheets (Figure 6B). This structure is common to all BCCP proteins or domains whose structures have been determined (Athappilly and Hendrickson 1995; Yao et al. 1997; Reddy et al. 2000; Broussard et al. 2013a) (Figure 7), and it is also shared by the lipoyl‐carrier domain of such enzymes as pyruvate dehydrogenase (Green et al. 1995; Berg and de Kok 1997; Howard et al. 1998) and 2‐oxoglutarate dehydrogenase (Berg et al. 1996; Ricaud et al. 1996). In both biotinylated and lipoylated enzymes, the prosthetic group is covalently bound to the side chain of a Lys residue that is similarly positioned in a hairpin turn between two β‐turns, associated with one of the flattened β‐barrel structures formed by two four‐stranded β‐sheets. This structural similarity may be associated with the mechanistic similarity between biotinylated and lipoylated enzymes, in which the prosthetic group sequentially visits multiple distinct active sites and thereby chemically links half‐reactions to achieve the overall chemical catalysis. Alternatively, the common structural features may be associated with the need to identify the Lys residue that is targeted for biotinylation by biotin ligase (Cronan 2001) or lipoylation by lipoate protein ligase (Green et al. 1995; Roberts et al. 1999).

The determined structure of the C‐terminal domain of the plant BCCP1 protein aligns very closely with the AlphaFold2‐generated predicted structure of this protein (Cα RMSD of 0.491 Å). More significantly, the experimentally determined structure of the plant C‐terminal domain of BCCP1 aligns significantly with the homologous domain of the *E. coli* BCCP, with Cα RMSD of 0.684 Å (Figure 7A). Moreover, the core of the plant and *E. coli* BCCP proteins, defined by the 2 β‐sheet domains, is also common to BCCP domains of other biotinylated enzymes (Figure 7B). One distinguishing feature among these BCCP domains is the inclusion of a “thumb”‐like protrusion that was first noted with the *E. coli* bcCP (Athappilly and Hendrickson 1995; Yao et al. 1997; Reddy et al. 2000; Broussard et al. 2013a), which also occurs in the structure of the plant bcCP1. The functional importance of this protrusion was validated by mutations of the conserved Tyr and Pro residues in this thumb‐like structure, which disrupts functionality of the *E. coli* BCCP (Cronan 2001). Figure 7B illustrates that this protrusion is absent from the BCCP domains of the human ACC2 (PDB ID: 2KCC) (Lee et al. 2008), the 1.3S subunit of the *P. shermanii* transcarboxylase (PDB ID: 1DCZ) (Reddy et al. 2000), methylmalonyl‐CoA decarboxylase (PDB ID: 2D5D) (Bagautdinov et al. 2008), pyruvate carboxylase (PDB ID: 5KS8) (Choi et al. 2016), and propionyl‐CoA carboxylase (PDB ID: 6YBP) (Scheffen et al. 2021), even though the core structure of the bcCP domains (i.e., the 2 β‐sheet domain) is common to all seven proteins.

### 3.3 Chemical Shift Mapping Experiments of Potential Protein–Protein Interactions Involving bcCP1

Multisubunit biotin enzymes, such as the htACCases, have to exhibit intermolecular physical interactions among subunits in order to assemble a functional holoenzyme. This has been directly demonstrated with the *E. coli* ACCase, with the assembly, for example, of a subcomplex between the bc and bcCP subunits (Broussard et al. 2013a). In the case of the plant htACCase, however, previous studies have shown that the assembly of such a bc‐bcCP subcomplex is facilitated by a noncatalytic subunit, BADC, which acts as a “glue” to assemble the holoenzyme complex (Salie and Thelen 2016; Shivaiah et al. 2020). The Arabidopsis genome encodes three BADC isozymes, and the BADC3 isozyme is most efficient in facilitating the assembly of this holoenzyme complex (Shivaiah et al. 2020).

**FIGURE 8:**
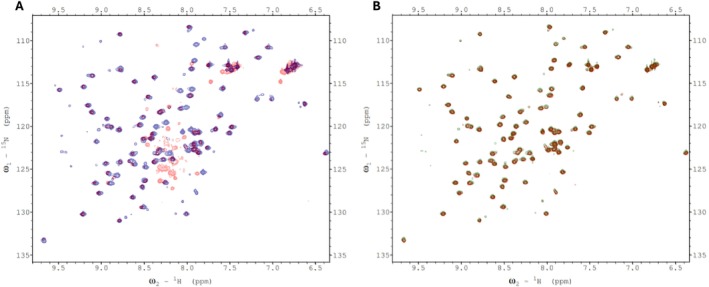
(A) Overlay of the 2D ^1^H‐^15^N HSQC spectra obtained with 100 μM ^15^N‐labeled mature BCCP1 in the absence of (blue) and presence of (red) 100 μM mature BC. (B) Overlay of the 2D ^1^H‐^15^N HSQC spectra obtained with 100 μM ^15^N‐labeled C‐terminal domain of BCCP1 in the absence of (green) and presence of (red) 100 μM mature BC subunit.

We therefore attempted to find evidence for such interactions for the plant htACCase by conducting chemical shift mapping experiments with ^15^N‐labeled bcCP1. Initially, we mixed either ^15^N‐labeled mature bcCP1 (Figure 8A) or ^15^N‐labeled C‐terminal domain of BCCP1 (Figure 8B) with purified Arabidopsis BC subunit. These chemical shift perturbation experiments were limited by the relative insolubility of the purified bc protein (BC aggregated at concentrations greater than 5 mg/mL). Indeed, dynamic light scattering experiments showed that multimers of BC were forming at these higher concentrations. Therefore the bcCP1:BC molar ratio could not be increased beyond 1:1. Despite this limitation, no evidence for the assembly of a bc‐BCCP1 complex was found in these mixing experiments. Consequently, further chemical shift perturbation experiments were conducted through copurification rather than by titrating individually purified subunits.

**FIGURE 9:**
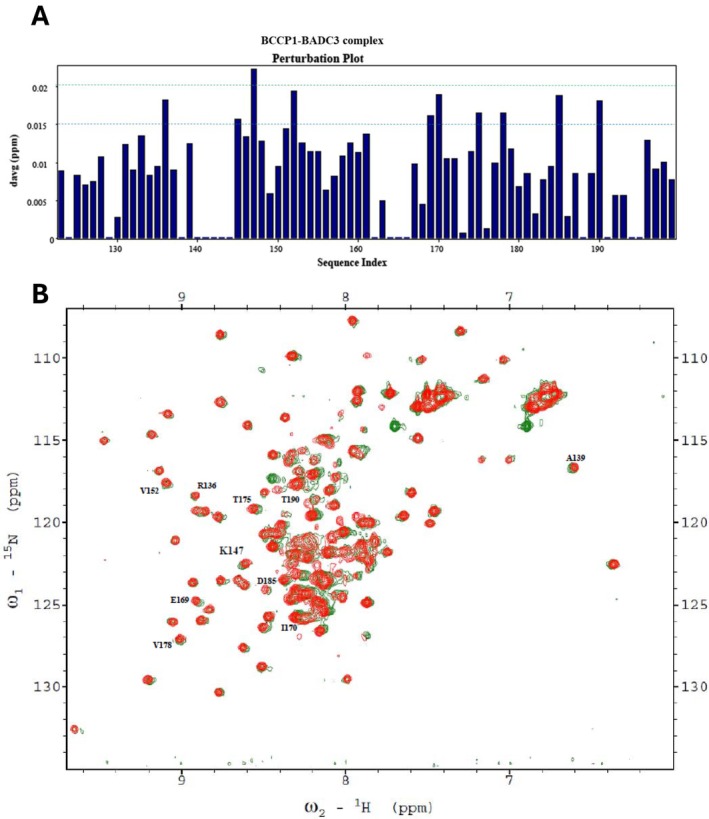
(A) Chemical shift perturbations of each residue of the C‐terminal domain of BCCP1 (130 μM) copurified with BADC3 (134 μM) in the absence of BC. Ten residues show a chemical shift perturbation, ΔδAvg_HN_ > 0.015 ppm. (B) The 2D ^1^H‐^15^N HSQC spectra of 130 μM ^15^N‐labeled mature BCCP1 (red), which was copurified with 134 μM unlabeled mature BADC3 (green). The residues showing a chemical shift perturbation (ΔδAvg_HN_ > 0.015 ppm) are identified.

**FIGURE 10:**
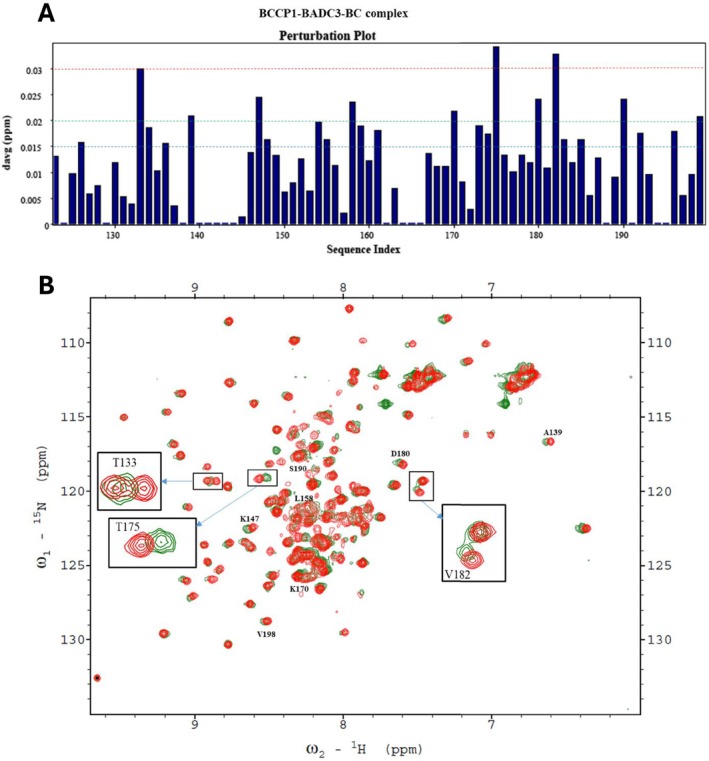
(A) Chemical shift perturbations of each residue of the C‐terminal domain of BCCP1 (114 μM) copurified with BADC3 (132 μM) and in the presence of BC (27 μM). Twenty‐four residues show a chemical shift perturbation, ΔδAvg_HN_ > 0.015 ppm. (B) The 2D ^1^H‐^15^N HSQC spectra of ^15^N‐labeled mature BCCP1 (114 μM) (red), which was copurified with unlabeled mature BC (27 μM) and mature BADC3 (132 μM) (green). The spectra of the three residues that show a chemical shift perturbation, ΔδAvg_HN_ > 0.03 ppm, are expanded, and the seven residues with the chemical shift perturbation of between ΔδAvg_HN_ > 0.02 and ΔδAvg_HN_ < 0.03 are also identified.

Chemical shift perturbation experiments were employed to explore potential protein–protein interactions between bcCP1, BADC3, and BC. We generated HSQC spectra with ^15^N‐labeled BCCP1 that was copurified with the BADC3 protein in the absence (Figure 9) and presence (Figure 10) of bc. When copurification was conducted with only the BADC3 protein, chemical shift perturbations of nine residues were detected, in the range ΔδAvg_HN_ 0.015–0.02 ppm (i.e., residues R136, F145, V152, E169, I170, T175, V178, D185, and S190), and only one residue (K147) showed a chemical shift perturbation value greater than 0.02 ppm (Figure 9A). This latter residue is located within the “thumb”‐like protrusion, which also occurs in *E. coli* ACCase (Athappilly and Hendrickson 1995; Yao et al. 1997; Reddy et al. 2000; Broussard et al. 2013a).

When all three proteins were copurified (i.e., ^15^N‐labeled bcCP1, BADC3, and bc), more significant changes in the chemical perturbations were observed (Figure 10A). Four of the nine residues that showed chemical perturbations with only BADC3 (in the absence of BC) showed more intense chemical shifts upon the inclusion of BC. These residues were K147, I170, T175, and S190, and their chemical shift perturbations increased by between 15% and 100%. Furthermore, six additional residues (T133, A139, L158, D180, V182, and V198) showed significant chemical shift perturbations (ΔδAvg_HN_ > 0.02 ppm) in the presence of both BADC3 and bc (Figure 10A). These chemical shift perturbation data are consistent with our prior genetic and biochemical reconstitution experiments, which indicated that BADC proteins facilitate interactions between bc and bcCP subunits.

**FIGURE 11:**
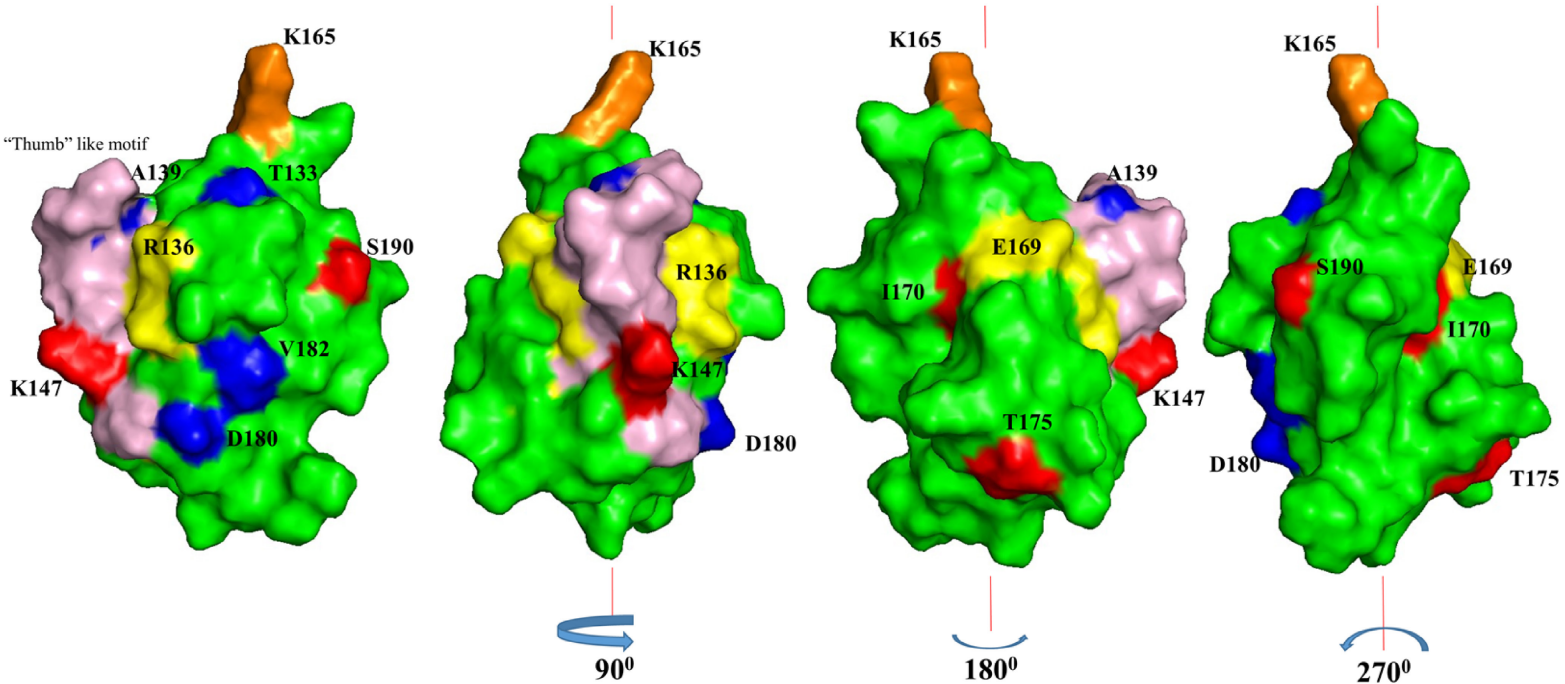
Mapping on the surface of the C‐terminal domain of BCCP1, the location of residues that show significant chemical shift perturbations upon the formation of the BC–BCCP1–BADC3 complex. The yellow‐highlighted residues displayed chemical shift perturbations with only BADC3. The blue‐highlighted residues displayed chemical shift perturbations in the presence of both BADC3 and BC, but not when BCCP1 was copurified with just BADC3. The red‐highlighted residues displayed increased chemical shift perturbation when BC is added to the copurified BCCP1 and BADC3. The residues that constitute the “thumb”‐like motif are highlighted in pink, and the biotinylated lysine‐165 residue is highlighted in orange.

Figure 11 shows the spatial location of the residues that show chemical shift perturbations, on the structure of the C‐terminal domain of bcCP1. With the exception of residue L158, all the residues that display chemical shift perturbations are located on the surface of the BCCP1 protein. These surface residues are concentrated on the side of the BCCP1 C‐terminal domain that contains the thumb‐like protrusion. We hypothesize therefore that this molecular surface may facilitate the intermolecular interactions that enable the formation of a complex between BCCP1 with BADC3 and/or BC.

It is informative to compare and contrast these suppositions for the plant htACCase with the findings that have been reported with the homologous bacterial enzyme. The BC–BCCP subcomplex of the *E. coli* ACCase is assembled via weak, ionic, and hydrogen bonds, with no observed hydrophobic interactions (St. Maurice et al. 2007; Lietzan et al. 2011; Broussard et al. 2013a). The residues participating in these interactions are located on the surface of BCCP that is opposite from the thumb‐like protrusion (Broussard et al. 2013a). Thus, if this bacterial structural organization can be used as a model for the plant htACCase subcomplex, one can envision that bc–BCCP1 interaction is stabilized by the binding of the BADC proteins, which may act as a “cap” to lock into place the BC–BCCP1 subcomplex. Moreover, although the thumb‐like protrusion is not directly involved in assembling the *E. coli* BC–BCCP subcomplex, this protrusion protein via dimerization (Cronan 2001). Therefore, it is plausible to suggest that in the plant htACCase, the BADC proteins stabilize the BC–BCCP1–BADC subcomplex, which is consistent with our prior in vitro reconstitution studies and in vivo genetic characterizations, which have indicated that the formation of such a subcomplex facilitates htACCase catalysis (Shivaiah et al. 2020) . However, it has also been suggested that BADC proteins may replace the BCCP protein from the htACCase complex and thereby inhibit catalytic activity (Salie et al. 2016; Keereetaweep et al. 2018). Because this inhibitory model does not necessitate binding between BADC and BCCP, we suggest that the structural constraints revealed by the current study are inconsistent with the inhibitory function for the BADC proteins. Hence, the plant structural model for the Arabidopsis BCCP1 presented herein provides valuable insight on the evolutionary relationship between htACCase in higher plant plastids and bacterial ACCase. Future mutagenesis research, coupled with the in vitro expression system that we developed for reconstituting the plant htACCase (Shivaiah et al. 2020), can evaluate the specific residues that the chemical shift perturbations suggest are involved in the assembly of the BC–BCCP1–‐BADC subcomplex.

## Author Contributions

K.‐K.S.: designed the research, performed the research, analyzed data, and wrote the paper.. A.W.B. and G.P.S.: contributed new analytic/computational/etc. tools and wrote the paper. B.J.N.: designed the research, performed research, contributed new analytic/computational/etc. tools, analyzed data, and wrote the paper.

## Disclosure

The content is solely the responsibility of the authors and does not necessarily represent the official views of the National Science Foundation.

## Conflicts of Interest

The authors declare no conflicts of interest.

## Peer Review

The peer review history for this article is available in the Supporting Information for this article.

## Supporting information


**Data S1** Peer Review.

**Table S1** The Rosetta energy scores (kilocalorie per mole) and RMSD (angstrom) values of the 10 best structures of BCCP1 with the lowest Rosetta energy scores.
**Figure S1**: Comparison of BCCP1 and BCCP2 sequences. The comparison was conducted using CLUSTAL OMEGA (ver. 1.2.4) (1). N‐terminal chloroplast transit peptide sequences are highlighted in turquoise. The C‐terminal domains that were the focus of this study are yellow‐highlighted, and the conserved biotinylated lysine residue is green‐highlighted. Identical residues are identified with an asterisk (*) below the sequences.
**Figure S2**: Plot of CS‐Rosetta energy score (kilocalorie per mole) versus Cα‐RMSD (angstrom) relative to lowest‐energy models for 3000 calculated structures of the C‐terminal domain of BCCP1.

## Data Availability

The datasets generated for this study can be found in the BioMagResBank database (http://www.bmrb.wisc.edu), as Accession BMRB ID 52087, and in wwPDB (https://www.wwpdb.org) as Accession PDBDEV_00000372.
