# Supplementary material for: Solution Structure and NMR Chemical Shift Perturbations of the Arabidopsis BCCP1 Identify Intersubunit Interactions Potentially Involved in the Assembly of the Heteromeric Acetyl‐CoA Carboxylase
Source: Plant Direct. 2025 Mar 21;9(3):e70057. doi: 10.1002/pld3.70057 (PMC11926652; doi:10.1002/pld3.70057)
Supplement: Supplementary file 2 — Table S1 The Rosetta energy scores (kilocalorie per mole) and RMSD (angstrom) values of the 10 best structures of BCCP1 with the lowest Rosetta energy scores. Figure S1: Comparison of BCCP1 and BCCP2 sequences. The comparison was conducted using CLUSTAL OMEGA (ver. 1.2.4) (1). N‐terminal chloroplast transit peptide sequences are highlighted in turquoise. The C‐terminal domains that were the focus of this study are yellow‐highlighted, and the conserved biotinylated lysine residue is green‐highlighted. Identical residues are identified with an asterisk (*) below the sequences. Figure S2: Plot of CS‐Rosetta energy score (kilocalorie per mole) versus Cα‐RMSD (angstrom) relative to lowest‐energy models for 3000 calculated structures of the C‐terminal domain of BCCP1. [file PLD3-9-e70057-s002.docx]

**SUPPLEMENTARY DATA:**

**Supplemental Table 1:** The Rosetta energy scores (kcal/mol) and rmsd (Å) values of 10 the best structures of BCCP1 with lowest Rosetta energy scores.

| Structure name | Rosetta energy score (kcal/mol) | C_α_-RMSD (Å) |
| --- | --- | --- |
| S_1007 | -145.986 | 0.2374 |
| S_2229 | -145.056 | 0.6999 |
| S_0209 | -144.603 | 0.2545 |
| S_01526 | -144.459 | 0.2271 |
| S_02580 | -144.047 | 0.3461 |
| S_00719 | -143.859 | 0.1864 |
| S_02636 | -143.783 | 0.2412 |
| S_00111 | -143.549 | 0.376 |
| S_00466 | -143.457 | 0.2525 |
| S_02291 | -143.306 | 0.4758 |
| Average C_α_-RMSD | N/A | 0.33 ± 0.16 |

BCCP1 MASSSFSVTSPAAAASVYAVTQTSSHFPIQNRSRRVSFRLSAKPKLRFLSKPSRSSYPVV 60

BCCP2 MASLSV----PC--------------VKICALNRRVG-SLPGISTQRWQPQPNGISFPSD 41

*** *. *. . * .***. * . . *: :*. *:*

BCCP1 KAQSNKVS-------TGASSNAAKVDGPSSAEGKEKNSLKESSASSPELATEESISEFLT 113

BCCP2 VSQNHSAFWRLRATTNEVVSNSTPMTNGGYMNGKA----------KTNVPEPAELSEFMA 91

:*.:.. . . **:: : . . :** . :: .:***::

BCCP1 QVTTLVKLVDSRDIVELQLKQLDCELVIRKKEALPQPQAPASYVMMQQPNQPSYAQQMAP 173

BCCP2 KVSGLLKLVDSKDIVELELKQLDCEIVIRKKEALQQAVPPAPVYHSMPPV---MADFSMP 148

:*: *:*****:*****:*******:******** * ** * *: *

BCCP1 PAAPAAAAPAPSTPASLPPPSPPTPAKSSLPTVKSPMAGTFYRSPAPGEPPFIKVGDKVQ 233

BCCP2 PAQPVALPPSPTPTSTPATAKPTSAPSSSHPPLKSPMAGTFYRSPGPGEPPFVKVGDKVQ 208

** *.* *:*: :: .* : .** * :************.******:*******

BCCP1 KGQVLCIVEAMKLMNEIESDHTGTVVDIVAEDGKPVSLDTPLFVVQP 280

BCCP2 KGQIVCIIEAMKLMNEIEAEKSGTIMELLAEDGKPVSVDTPLFVIAP 255

***::**:**********::::**:::::********:******: *

**Supplemental Figure S1**: Comparison of BCCP1 and BCCP2 sequences. The comparison was conducted using CLUSTAL OMEGA (ver. 1.2.4) (*1*). N-terminal chloroplast transit peptide sequences are highlighted in turquoise. The C-terminal domains that were the focus of this study are yellow-highlighted, and the conserved biotinylated lysine residue is green-highlighted. Identical residues are identified with an asterisk (*) below the sequences.

1. F. Madeira *et al.*, Search and sequence analysis tools services from EMBL-EBI in 2022. *Nucleic Acids Res* **50**, W276-W279 (2022).


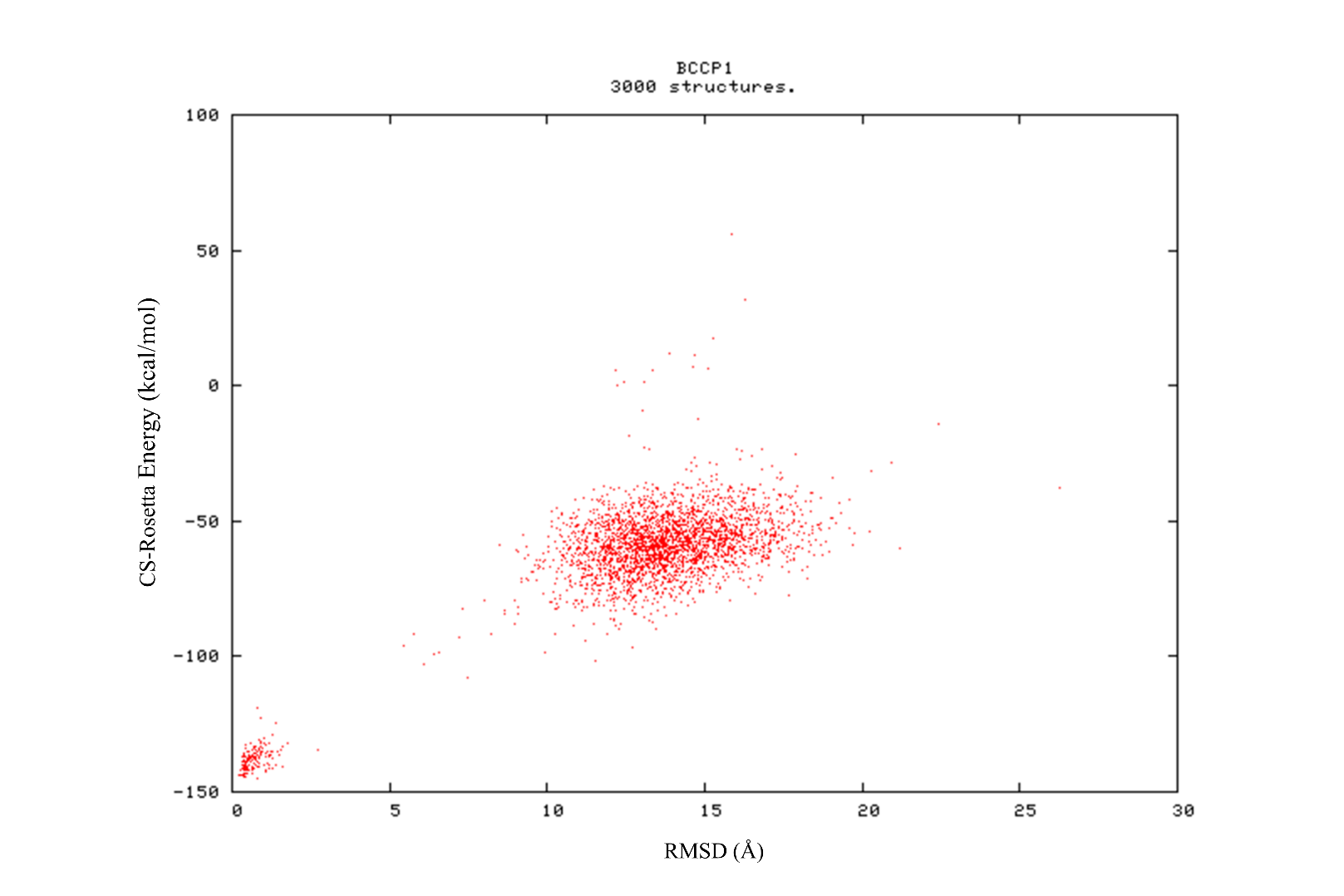


**Supplemental Figure S2**: Plot of CS-Rosetta energy score (kcal/mol) vs Cα-RMSD (Å) relative to lowest-energy models for 3000 calculated structures of the C-terminal domain of BCCP1.
