# Supplementary material for: Solution Structure and NMR Chemical Shift Perturbations of the Arabidopsis BCCP1 Identify Intersubunit Interactions Potentially Involved in the Assembly of the Heteromeric Acetyl‐CoA Carboxylase
Source: Plant Direct. 2025 Mar 21;9(3):e70057. doi: 10.1002/pld3.70057 (PMC11926652; doi:10.1002/pld3.70057)
Supplement: Supplementary file 1 — Data S1 Peer Review. [file PLD3-9-e70057-s001.pdf]

Plant Direct
Open Access

Solution structure and NMR chemical shift perturbations of the Arabidopsis BCCP1 identify inter-subunit interactions potentially involved in the assembly of the heteromeric acetyl-CoA carboxylase

Kiran-Kumar Shivaiah,[1,2,3,#] Ganesh P. Subedi,[1] Adam W. Barb,[1,2,@] Basil J. Nikolau, [1,2,3,*]

Decision Letter Round 1:

January 14, 2025

Dr. Basil J. Nikolau
Iowa State University of Science and Technology
Biochemistry, Biophysics and Molecular Biology
Ames, Iowa 50011

RE: Solution structure and NMR chemical shift perturbations of the Arabidopsis BCCP1 identify inter-subunit interactions potentially involved in the assembly of the heteromeric acetyl-CoA carboxylase

Dear Dr. Nikolau:

Thank you for submitting to Plant Direct. All required reviews have been returned and we have now finished our evaluation of your manuscript. In light of the reviewers' and editor's comments, further revisions are needed before the paper can be accepted for publication in Plant Direct.

Please view the editors' and reviewers' comments below and use their suggestions as a guide while you work on your revision.

When uploading the revised version of this article, please be sure to include the following:

-A word document that contains your response to the reviewers. You should respond to each reviewer comment and note the changes made to the manuscript. If you do not agree with a reviewer's comment and choose not to make a suggested revision, please explain why. Please try to provide as complete an answer as possible to each reviewer's criticisms.
-A tracked changes document with each change highlighted
- A clean version of the latest version of the manuscript

Sorry for the wait Basil, and thank you for your patience. Esther

Plant Direct
Open Access

To upload your revision, please click the link below.
https://plantdirect.msubmit.net/cgi-bin/main.plex?el=A5Lr5kt4A5cs2I4A9ftdGRRMghLraeGH93L9i1XQZ

In order to provide as timely a service as possible, we ask that your revision is resubmitted within three months after receipt of this request. If an extension is needed, please send a request, along with a brief explanation, to the editorial office at plantdirect@wiley.com .

Please note that, in addition to publishing reviewer comments, the author's responses to review comments will also be published alongside the final version of the paper. If you would not like the author's responses to be published, please contact the editorial office at plantdirect@wiley.com .

Thank you very much for giving us an opportunity to review your work. I look forward to receiving the next version.

Sincerely,

Esther van der Knaap

Esther van der Knaap

Editor, Plant Direct

-- Editor comments:
Sorry for the wait Basil, and thank you for your patience. Esther

-- Reviewer comments:
Reviewer #1:

The authors present solution structure and NMR chemical shift perturbations of the Arabidopsis BCCP1 to identify inter-subunit interactions potentially involved in the assembly of the heteromeric acetyl-CoA carboxylase. The chemical shift perturbation experiments identified potential surface residues on the BCCP1 protein that may facilitate physical interactions between BC and BADC proteins. The paper is interesting, the results are technically of good level, however, some minor revisions are requested before acceptance.
1. Page 7. Lines 181-185. "The chemical shift perturbation values (ΔδAvg HN) for the backbone amide bonds were evaluated by using NMRSPARKY program. The equation used for this calculation was:

ΔδAvg HN √((ΔN)^2+(ΔN/2))^2

Where, ΔH and ΔN are the chemical shift changes of the amide 1H and 15N in ppm, induced by conformational changes upon protein-protein interactions".

Chemical shift perturbations of 13C NMR are also a very useful indication of structural motifs and conformational changes upon protein-protein interactions. The authors should also utilize 13C chemical shifts in their analysis of NMR data.

2. Figure 1. Some critical assignments should be presented.

3. Figures 3 and 7. Should be significantly improved.

x

Reviewer #2:

This is a review of the article titled "Solution structure and NMR chemical shift perturbations of the Arabidopsis BCCP1 identify inter-subunit interactions potentially involved in the assembly of the heteromeric acetyl-CoA carboxylase" by Shivaiah and coworkers. I am a plant lipid biochemist, and although I am not an expert in NMR, I can readily appreciate the significance and importance of the author's work. Studies of this type are needed to fill major gaps in our knowledge. One of the major conclusions of this study is the identification of surface residues on BCCP1 that mediate interactions with BC and BADC. This conclusion is supported by chemical shift perturbation experiments and provides clear future directions for study. Overall, this manuscript is well written, the rationale for the study is sound, it is interesting, and the study is well grounded in existing literature, both in terms of the rationale and in terms of the findings presented. I have a few minor suggestions that I detail below. I suggest that the authors consider these suggestions, and I thank them for their work in this important area.

Minor comments:

1. I think that some readers may benefit from a diagram early in the article, perhaps in the introduction, that illustrates how the different components being described in this article fit (or are hypothesized to fit, since such is the topic of investigation here) together. For example, BCCP, htACCase, Biotin, BC, and which constitute the holoenzyme, which are BADC proteins, and so forth. I realize that Figure 10 to some degree accomplishes this suggestion, but I think that some type of schematic early on would help keep readers oriented when the many different macromolecules being described are introduced.

2. I am not well versed in the literature describing the relationship between BADC proteins and htACCase. However, based on the present work's discussion and the references cited, it seems like there is some inconsistency or dispute in this area. Perhaps there is not much more that can be added to the present discussion to provide further detail on these inconsistencies. However, if it is possible to describe these in further detail and outline the competing hypotheses and the evidence for or against each in more detail, such modifications may help readers think more holistically about the system being studied.

Plant Direct
Open Access

Decision Letter Round 2:

March 2, 2025
Dr. Basil J. Nikolau
Iowa State University of Science and Technology
Biochemistry, Biophysics and Molecular Biology
Ames, Iowa 50011

MSID: 2024-01629R1
MS TITLE: Solution structure and NMR chemical shift perturbations of the Arabidopsis BCCP1 identify inter-subunit interactions potentially involved in the assembly of the heteromeric acetyl-CoA carboxylase

Dear Dr. Basil Nikolau:

I am pleased to inform you that your manuscript "Solution structure and NMR chemical shift perturbations of the Arabidopsis BCCP1 identify inter-subunit interactions potentially involved in the assembly of the heteromeric acetyl-CoA carboxylase" has been accepted for publication in Plant Direct. Thank you for your thoughtful replies to reviewers and needed updates.

Your article will appear online in the next available issue of Plant Direct. To ensure your article gets published as quickly as possible, please pay attention to the steps detailed below. We have found that most of the delays happen at this stage, especially at the payment stage, so please respond as quickly as possible when prompted.

License Agreement: Once your article has been accepted it will move to Production and undergo admin and file checking - you may receive an email with any queries we have at this stage. When all required items are received by the publisher and queries resolved, the corresponding author will receive an email from Wiley's Author Services system which will ask them to log on at https://authorservices.wiley.com/bauthor and will present them with the appropriate license for completion. Your article cannot be published until both the signed license agreement and payment of the article fee have been received.

Payment of the Open Access Article Publication Fee: All articles published in Plant Direct are fully open access: immediately and freely available to read, download and share. Plant Direct charges a publication fee to cover the publication costs. The corresponding author for this manuscript should have already received a quote from the payments team (cs-openaccess@wiley.com) with the estimated article publication fee; please email cs-openaccess@wiley.com if this has not been received. The corresponding

author should log on to the Wiley Author Services site, where the publication fee can be paid by credit card or an invoice. Pro Forma can also be requested. Payment of the publication charge must be received before the article will be published online.

Proofs: You will have the opportunity to look over your paper once more when you receive the author proofs for your article. The proofs will be with you in approximately two weeks. Please note that, in addition to publishing reviewer comments, the author's responses to review comments will also be published alongside the final version of the paper. If you would not like the author's responses to be published, please contact the editorial office at plantdirect@wiley.com .

Promotion of your article: You can help your research get the attention it deserves! Check out Wiley's free Promotion Guide for best-practice recommendations for promoting your work at www.wileyauthors.com/eeo/guide. And learn more about Wiley Editing Services which offers professional video, design, and writing services to create shareable video abstracts, infographics, conference posters, lay summaries, and research news stories for your research at www.wileyauthors.com/eeo/promotion.

Thank you again for your contribution to Plant Direct. If you have any questions, feel free to contact the editorial office at plantdirect@wiley.com .

Sincerely,

Larry York

EIC, Plant Direct

Response to Reviewer:

TO: Dr. Esther van der Knaap
Editor, *Plant Direct*

RE: Revision of 2024-01629

Dear Esther,

We are resubmitting for your consideration the manuscript entitled, *"Solution structure and NMR chemical shift perturbations of the Arabidopsis BCCP1 identify inter-subunit interactions potentially involved in the assembly of the heteromeric acetyl-CoA carboxylase"*, which was reviewed January 14, 2025. Thank you for your diligence in reviewing this manuscript.

Plant Direct
Open Access

The manuscript has been revised in response the each of the reviewer's comments and suggestions, and these are specifically identified below. We have also uploaded a revised manuscript in which our edits have been identified by the Tracked Changes function of Word.

Please let me know if

Sincerely,

Basil J. Nikolau,
Frances M. Craig Professor Emeritus

Reviewer #1:

1) *Reviewer's comment: Chemical shift perturbations of ^{13}C NMR are also a very useful indication of structural motifs and conformational changes upon protein-protein interactions. The authors should also utilize ^{13}C chemical shifts in their analysis of NMR data.*

Authors' response: We agree with the reviewer that chemical shift perturbations data of ^{13}C NMR are also of utility in revealing confirmational changes upon protein-protein interactions, unfortunately such data were not gathered in this study.

2) *Reviewer's comment: Figure 1. Some critical assignments should be presented.*

Authors' response: As requested, we have revised Figure 1, and identified assignments of critical residues.

1) Reviewer's comment: *Figures 3 and 7. Should be significantly improved.*

Authors' response: As requested, we have revised Figure 3 and 7, and increased the font sizes of the x- and y-axes labels, which improves the quality of these two figures.

Reviewer #2:

Minor comments:

1) Reviewer's comment: I think that some readers may benefit from a diagram early in the article, perhaps in the introduction, that illustrates how the different components being described in this article fit (or are hypothesized to fit, since such is the topic of investigation here) together. For example, BCCP, htACCase, Biotin, BC, and which constitute the holoenzyme, which are BADC proteins, and so forth. I realize that Figure 10 to some degree accomplishes this suggestion, but I think that some type of schematic early on would help keep readers oriented when the many different macromolecules being described are introduced.

Authors' response: As requested we have included a new Figure 1, which shows a schematic of the quaternary organization of the heteromeric ACCase, and cited this figure in the appropriate text of the Introduction. To better accommodate this new figure, we have rearranged text in the second and third paragraph of the Introduction.

2) Reviewer's comment: I am not well versed in the literature describing the relationship between BADC proteins and htACCase. However, based on the present work's discussion and the references cited, it seems like there is some inconsistency or dispute in this area. Perhaps there is not much more that can

be added to the present discussion to provide further detail on these inconsistencies. However, if it is possible to describe these in further detail and outline the competing hypotheses and the evidence for or against each in more detail, such modifications may help readers think more holistically about the system being studied.

Authors' response: The reviewer is correct concerning inconsistency in the literature concerning the function of the BADC proteins. We had previously extensively provided an explanation as to how we believe this inconsistency arose in our prior publication (citation Shivaiah et al., 2020). We have explicitly stated this inconsistency in the last paragraph of the Introduction.
